# Impact of Early Rehabilitation on Outcomes in Patients With Acute Ischemic Stroke After Endovascular Treatment

**DOI:** 10.3389/fneur.2022.877773

**Published:** 2022-05-23

**Authors:** Yi He, Ximing Nie, Tao He, Xiao Qi, Zhenzhen Chen, Wei Duan, Yufei Wei, Xiran Liu, Yong Liu

**Affiliations:** ^1^Department of Pain and Rehabilitation, Xinqiao Hospital, Army Medical University, Chongqing, China; ^2^Department of Neurology, Beijing Tiantan Hospital, Capital Medical University, Beijing, China; ^3^Department of Neurology, Xinqiao Hospital, Army Medical University, Chongqing, China

**Keywords:** early rehabilitation, acute ischemic stroke, large vessel occlusion stroke, endovascular treatment, functional outcome

## Abstract

**Background:**

This study aims to examine the effects of early rehabilitation on functional outcomes in patients with acute ischemic stroke treated with endovascular treatment (EVT).

**Methods:**

Eligible patients with large vessel occlusion stroke treated with EVT, who received early rehabilitation or standard care treatment during hospitalization, were enrolled in a multicenter registration, prospective observational study, a registration study for Critical Care of Acute Ischemic Stroke After Recanalization. Early rehabilitation was defined as rehabilitation interventions initiated within 1 week after acute stroke. The primary outcome was the favorable functional outcome (defined as modified Rankin Scale scores of 0 to 2) at 90 days. Independent association between early rehabilitation and the primary outcome was investigated using multivariable logistic regression in the entire sample and in subgroups.

**Results:**

A total of 1,126 patients (enrolled from July 2018 to May 2019) were included in the analyses, 273 (24.2%) in the early rehabilitation group and 853 (75.8%) in the standard care group. There was no significant difference in favorable functional outcomes at 90 days between the two groups (45.4 vs. 42.6%, *p* = 0.41). Patients in the early rehabilitation group had a lower death rate within 90 days compared with the standard care group (6.2 vs. 20.5%, *p* < 0.01). The multivariable logistic regression analyses showed that the early rehabilitation was not significantly associated with the favorable functional outcome at 90 days (adjusted odds ratio, 1.01 [95% CI, 0.70–1.47]; *p* = 0.95). There was no significant difference between subgroups in the favorable functional outcome at 90 days. No significant interaction was found between subgroups.

**Conclusions:**

Patients with stroke receiving early rehabilitation had a lower death rate. However, these clinically meaningful effects of early rehabilitation did not show on functional outcome at 90 days in patients with large vessel occlusion stroke treated with EVT.

**Registration:**

URL: http://www.chictr.org.cn; Unique identifier: ChiCTR1900022154.

## Introduction

Stroke is the leading cause of long-term disability in developed countries and one of the top causes of death worldwide (1). Endovascular treatment (EVT) is widely recognized as an effective method for acute ischemic stroke (AIS) due to large vessel occlusion (LVO) and has been recommended in the latest guidelines for AIS (2). Advances in acute stroke management have improved the survival of patients with AIS by providing better functional outcomes. However, many survivors still experience persistent difficulty with daily tasks. Data suggest that more than half of the patients receiving EVT would still suffer from disability at 90 days (3) and approximately 15% of them would still have severe disability at 90 days (4). Thus, an effective stroke rehabilitation program is an essential component of stroke care.

Guidelines have recommended early mobilization after stroke to improve activities of daily living (2, 5–7). Data strongly suggest that there are benefits to starting rehabilitation as soon as the patient is ready and can tolerate it (8, 9). However, the optimal time to begin rehabilitation after a stroke remains unsettled. A randomized controlled study in A Very Early Rehabilitation Trial for stroke (AVERT) concluded that commencing intensive therapy in the first 24 h may be harmful (10). The evidence is mounting that initiation of rehabilitative strategies within the first 2 weeks of stroke is beneficial (11). A literature review showed inconsistent results for the effects of early rehabilitation vs. delayed rehabilitation (12). Research on early rehabilitation in patients with AIS after EVT is relatively sparse, largely due to logistical challenges in the acute phase, including medical instability (13–15), activity restrictions and comorbidities in many patients with acute stroke, and the difficulty of conducting research across intensive care units.

This study examined the effect of early rehabilitation on long-term functional outcomes in patients with LVO stroke after EVT, using the database of a national clinical research center for neurological diseases in China.

## Methods

### Source of Data

The database was developed by a registration study for Critical Care of Acute Ischemic Stroke After Recanalization (RESCUE-RE). RESCUE-RE is a multicenter, prospective observational study that has been completed. The clinical database was developed based on data from 18 comprehensive stroke centers across China. The aim of RESCUE-RE was to evaluate the short- and long-term outcomes of patients with AIS who received EVT in the real-world clinical practice.

In RESCUE-RE, data from individual patients were systematically collected in each center and clinical databases were collected through China National Clinical Research Center for Neurological Diseases at http://ctms.tt.zhinanmed.com. Baseline clinical information and outcomes were collected by trained research coordinators. Compilation of completed forms from all centers was examined by full-time study-quality coordinators.

### Participants

In this real-world clinical practice study, the clinical data of patients during the period between July 2018 and May 2019 were collected from the China National Clinical Research Center for Neurological Diseases. All patients met the following criteria: (1) age>18 years, (2) AIS confirmed by cerebral imaging, and (3) treatment with recanalization for AIS according to the current guidelines (2). The study excluded patients who (1) lacked rehabilitation data, (2) could not perform independent daily activities before stroke (modified Rankin Scale [mRS] score>2), and (3) were lost to follow up at 90 days. All participants were admitted to the intensive care unit after EVT and managed according to the latest guidelines for AIS^(2)^.

### Early Rehabilitation and Standard Care Treatment

The rehabilitation group received early rehabilitation and the standard care treatment. Early rehabilitation is defined as a specialized poststroke rehabilitation program administered by physicians, physical therapists, and occupational therapists within 1 week after acute stroke (7, 11). The rehabilitation plan includes following activities: sitting supported in bed, sitting unsupported out of bed, transfer along with assistance, roll and sit up, sitting without support, transfer feet on the floor, standing activities, walk-early gait, advanced gait activities (10, 16), and imitate what would occur in normal activities of daily living or clinical practice (17). The intensity of rehabilitation is usually 45 min a day, 5 to 7 days per week. Rehabilitation plan and intensity was adjusted dynamically according to the patients' needs and abilities. The rehabilitation period lasted 14 days or until discharge from stroke unit care, whichever was sooner.

Close monitoring of the blood pressure and heart rate was necessary during the rehabilitation procedure, immediately after and 5 min after, while patients showing any sign of low tolerance, defined by neurological worsening (of current or new neurological deficits), vagal reaction (bradycardia or nausea), a >40 mmHg increase of blood pressure topping 180/100 mmHg, or a symptomatic decrease in blood pressure, would be put back in bed (18).

Patients in the standard care group received routine stroke unit care, including the passive and active (if possible) mobilization, correct positioning in bed, sitting balance activities, facilitation of limb and trunk control activities, and education of patient and caregiver. Both groups received standard care treatment, for 45 min a day, for 14 days, or until discharge (16).

### Outcomes

The patient characteristics were prospectively collected in all participating centers. Successful recanalization was defined as a thrombolysis in cerebral infarction (TICI) score of 2b or 3 after EVT (3). The primary outcome was favorable functional recovery at 90 days after the onset of stroke, defined as an mRS score of 0 to 2 (10, 19). Secondary outcomes were (1) mortality at 90 days defined as all-cause death within 90 days, (2) length of hospital stay, defined as the number of days in the hospital, (3) stroke severity assessed by the National Institute of Health Stroke Scale (NIHSS) on discharge (20), and (4) cost in hospitalization, defined as the expenses of all medications, surgeries, and medical consumables during hospitalization. Outcomes at 90 days were assessed during outpatient visits or by telephone.

### Statistical Methods

The demographic and clinical characteristics were compared between the early rehabilitation group and the standard care group using χ^2^ tests or Fisher's exact test for dichotomous variables and Student's *t*-tests or Mann–Whitney U test for continuous variables. The level of significant difference was set at *p* < 0.05. Multivariate logistic regression analyses were performed to evaluate the relationship between favorable functional recovery (mRS 0–2) and the implementation of rehabilitation. These independent variables were chosen based on their clinical significance, previous studies (19, 21, 22) and univariable analyses (variable with *p* < 0.05). Model 1 was adjusted for age, sex, diabetes mellitus, hypertension, atrial fibrillation, dyslipidemia, smoking, education, and insurance. In model 2, time from stroke onset to admission interval, mRS before the stroke, baseline NIHSS score, NIHSS score at 24 h after EVT, the side of the brain where the stroke occurred, and stroke cause were added. In model 3, successful recanalization, defined as a TICI score of 2b or 3, was further added. Subgroup analyses were performed to detect heterogeneity in rehabilitation's effects on the primary outcome with statistical tests of interactions. All statistical analyses were performed using SAS software v 9.4 (SAS Institute, Inc, Cary, NC).

### Ethics Approval and Study Registration

The study protocol was evaluated and approved by the medical ethics committee, number KY2014-045-03. All patients provided written informed consent to participate in the study. This trial was registered with the Chinese Clinical Trial Registry, number ChiCTR1900022154.

## Results

### Baseline Characteristics

**Figure 1:**
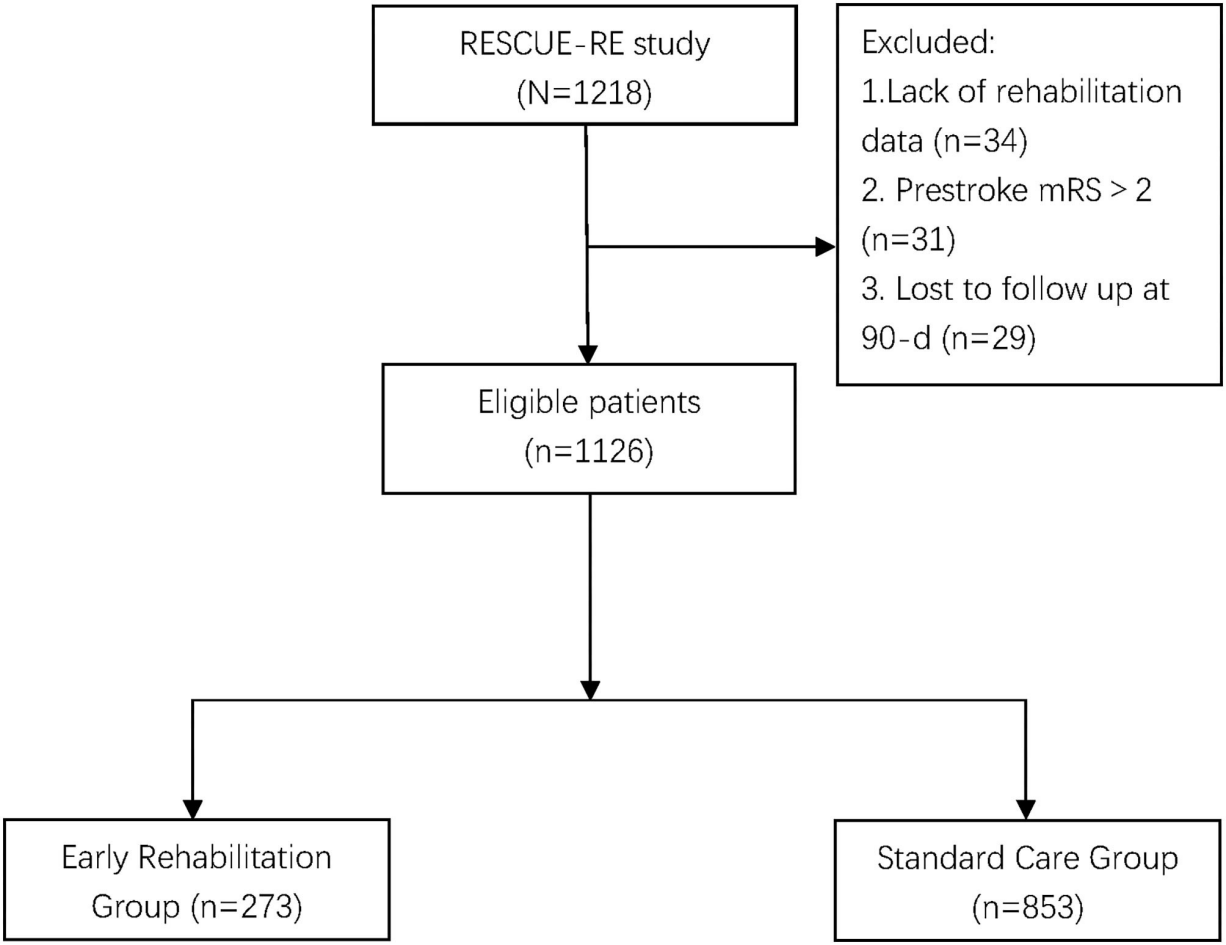
Participant selection flow chart. RESCUE-RE refers to a registration study for Critical Care of Acute Ischemic Stroke After Recanalization. mRS: modified Rankin Scale.

A total of 1,218 patients with AIS treated with EVT were enrolled in RESCUE-RE. Among them, 34 patients without rehabilitation therapy data, 31 patients with a prestroke mRS of > 2, and 29 patients lost to follow-up at 90 days were excluded. Data on 1,126 patients were analyzed, 273 (24.2%) in the early rehabilitation group and 853(75.8%) in the standard care group. Figure 1 shows the selection procedure of patients.

The baseline characteristics of the two groups are detailed in Table 1. The patients in the early rehabilitation group were significantly older and less likely to have dyslipidemia. Patients with a college degree and an mRS score of 0 before stroke were more likely to receive early rehabilitation. Patients with a better recanalization (assessed by residual stenosis) after EVT were more likely to receive early rehabilitation. No significant differences were found between the two groups in sex, body mass index (BMI), NIHSS at admission and 24 h after EVT, the side of the brain where the stroke occurred, stroke cause, previous medication, TICI, time from stroke onset to admission interval, and complications.

**Table 1:** Baseline characteristics of the patients.

**Characters**	**Total**	**Early rehabilitation group**	**Standard care group**	***p*** **-value**
	**(*n* = 1,126)**	**(*n* = 273)**	**(*n* = 853)**	
Age, Mean ± SD	64.7 ± 12.2	66.6 ± 11.7	64.1 ± 12.3	0.00
Male, *n* (%)	721(64.1)	172(63.0)	549(64.4)	0.67
BMI, Mean ± SD	23.1 ± 4.2	22.2 ± 3.1	23.5 ± 4.5	0.18
**Medical history**, ***n*** **(%)**				
Diabetes mellitus	248(22.1)	58(21.3)	190(22.4)	0.70
Hypertension	645(57.4)	154(56.4)	491(57.7)	0.71
Atrial fibrillation	222(19.7)	61(22.3)	161(18.9)	0.21
Dyslipidemia	79(7.0)	11(4.0)	68(8.0)	0.03
Smoking	385(34.3)	101(37.0)	284(33.4)	0.27
**Education**, ***n*** **(%)**				0.23
College	108(9.6)	35(12.8)	73(8.6)	0.04
Senior high school	219(19.5)	58(21.3)	161(18.9)	0.38
Junior high School	285(25.4)	62(22.7)	223(26.2)	0.25
Elementary	196(17.4)	42(15.4)	154(18.1)	0.30
Illiteracy	37(3.3)	7(2.6)	30(3.5)	0.47
No clear	279(24.8)	69(25.3)	210(24.7)	0.85
**mRS before the stroke**, ***n*** **(%)**				0.00
0	1000(89.1)	256(94.1)	744(87.5)	0.002
1	99(8.8)	11(4.0)	88(10.4)	0.001
2	23(2.1)	5(1.8)	18(2.1)	0.76
Baseline NIHSS score, median (IQR)	16(12–21)	16(12–21)	16(12–20)	0.47
NIHSS 24h after EVT, median (IQR)	14(7–19)	14(8–17)	14(7–20)	0.53
**The side of the brain where the stroke occurred, n (%)**				0.41
Left	440(39.1)	109(39.9)	331(38.8)	
Right	504(44.8)	129(47.3)	375(44.0)	
Bilateral	95(8.4)	18(6.6)	77(9.0)	
**Stroke cause**, ***n*** **(%)**				0.15
Large-artery atherosclerosis	683(61.0)	154(56.6)	529(42.5)	
Cardioembolic	390(34.9)	103(37.9)	287(33.9)	
Others	46(4.1)	15(5.5)	31(3.7)	
**Artery occlusion**, ***n*** **(%)**				
ACA	38(3.4)	12(4.4)	26(3.1)	0.28
MCA	434(38.5)	115(42.1)	319(37.4)	0.16
CA	302(26.8)	74(27.1)	228(26.7)	0.90
PCA	31(2.8)	6(2.2)	25(2.9)	0.52
VA	88(7.8)	15(5.5)	73(8.6)	0.10
BA	150(13.3)	22(8.1)	128(15.0)	0.00
**Previous medication, n (%)**				
Antiplatelet therapy	235(21.6)	61(23.0)	174(21.2)	0.53
Antihypertensive therapy	403(78.4)	96(76.2)	307(79.1)	0.77
**Reperfusion therapy**, ***n*** **(%)**				
IVT	140(12.4)	45(16.5)	95(11.1)	0.02
Heparin during EVT	238(21.1)	54(19.8)	184(21.6)	0.53
Balloon dilatation	311(27.6)	66(24.2)	245(28.7)	0.16
Mechanical thrombectomy	1012(89.9)	248(90.8)	764(89.6)	0.54
**Reperfusion after intervention (TICI)**, ***n*** **(%)**				0.72
0–2a	235(20.9)	55(20.2)	180(21.2)	
2b−3	888(79.1)	218(79.9)	670(78.8)	
Residual stenosis, median (IQR)	1(0-30)	0(0-20)	5(0-30)	0.02
Time from stroke onset to admission interval, median (IQR), min	405(240–620)	387(233–600)	413(244–626)	0.20
**Complication**, ***n*** **(%)**				
Pneumonia	570(51.0)	148(54.2)	422(49.9)	0.22
Lower extremity deep vein thrombosis	130(11.6)	38(13.9)	92(10.9)	0.17
Pressure ulcers	2(0.18)	0(0.0)	2(0.2)	0.62

*IQR, interquartile range; mRS, modified Rankin Scale; NIHSS, National Institute of Health Stroke Scale; EVT, endovascular treatment; ACA, anterior cerebral artery; MCA, anterior cerebral artery; CA, carotid artery; PCA, posterior cerebral artery; VA, vertebral artery; BA, basilar artery; IVT, intravascular thrombolysis; TICI, thrombolysis in cerebral infarction*.

### Outcomes

**Figure 2:**
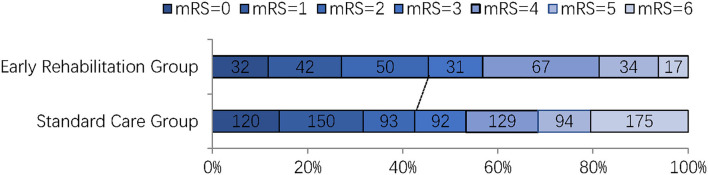
The mRS score at 3 months. mRS, modified Rankin Scale.

The outcomes of the two groups are detailed in Table 2. In total, 487 (43.3%) patients had favorable functional recovery (mRS 0–2) at 90 days, with a higher proportion in the early rehabilitation group; however, the difference was not statistically significant (45.4 vs. 42.6%; *p* = 0.41). The distribution of mRS at 90 days in two groups is presented in Figure 2. Patients in the early rehabilitation group had a lower death rate at 90 days compared with the standard care group (6.2 vs. 20.5%, *p* < 0.01). Length of hospital stay was longer in the early rehabilitation group, with a median of 301 h, compared with 225 h in the standard care group (*p*<0.01). There were no significant differences in other secondary outcomes, including NIHSS at discharge and medical cost during hospitalization (Table 2).

**Table 2:** Primary and secondary outcomes between groups.

	**Total (*n* = 1,126)**	**Early rehabilitation group (*n* = 273)**	**Standard care group (*n* = 853)**	***p***
**Primary outcome**				
mRS 0–2 at 90-d, *n* (%)	487(43.3)	124(45.4)	363(42.6)	0.41
Secondary outcome				
**Mortality at 90-d**, ***n*** **(%)**	192(17.1)	17(6.2)	175(20.5)	0.00
Length of hospital stay, h, median (IQR)	243(128–366)	301(165–439)	225(119–344)	0.00
NIHSS at discharge, median (IQR)	9(3–15)	9(3–14)	9(3–16)	0.44
Cost in hospital, RMB, Mean ± SD	105790.8 ± 63829.5	99188.7 ± 57190.3	107872.0 ± 65677.7	0.27

*mRS, modified Rankin Scale; IQR, interquartile range; NIHSS, National Institute of Health Stroke Scale; RMB: RenMin Bi,Yuan*.

In univariate analysis, early rehabilitation (OR, 1.12 [95% CI, 0.85–1.48]; *p* = 0.41) was not significantly associated with favorable functional recovery (mRS 0–2) at 90 days (Table 3). In multivariate logistic regression analyses, all models with adjustment for confounding factors showed that early rehabilitation was still not associated with favorable functional recovery (mRS 0–2) at 90 days. In multivariate logistic regression analyses for secondary outcomes, after adjusting for potential confounders in models 1, 2, and 3, these associations were still statistically significant (Table 3).

**Table 3:** Univariate and multivariate logistic regression of primary and secondary outcomes.

	**Univariate analysis**		**Multivariate analysis**					
			**Model 1** *		**Model 2**		**Model 3‡**	
	**OR (95% CI)**	***p*** **–value**	**OR (95% CI)**	***p*** **–value**	**OR (95% CI)**	***p*** **–value**	**OR (95% CI)**	***p*** **–value**
**mRS 0–2 at 90–d**								
Standard care	Reference							
Early rehabilitation	1.12(0.85–1.48)	0.41	1.14(0.85–1.52)	0.37	1.02(0.70–1.47)	0.93	1.01(0.70–1.47)	0.95
**Mortality at 90–d**								
Standard care	Reference							
Early rehabilitation	0.26(0.15–0.43)	<0.0001	0.24(0.14–0.40)	<0.0001	0.26(0.15–0.43)	<0.0001	0.24(0.14–0.40)	<0.0001
**Length of hospital stay**								
Standard care	Reference							
Early Rehabilitation	2.19(1.61–2.98)	<0.0001	2.34(1.69–3.24)	<0.0001	2.19(1.61–2.98)	<0.0001	2.34(1.69–3.24)	<0.0001

*OR, odds ratio; CI, confidence interval; mRS, modified Rankin Scale*.

* *Model 1 was adjusted for age, sex, diabetes mellitus, hypertension, atrial fibrillation, dyslipidemia, smoking, education, insurance*.

*Model 2 was adjusted for age, sex, diabetes mellitus, hypertension, atrial fibrillation, dyslipidemia, smoking, education, insurance, time from stroke onset to admission interval, mRS before the stroke, baseline NIHSS score, NIHSS score 24h after EVT, the side of the brain where the stroke occurred, and stroke cause*.

‡ *Model 3 was adjusted for age, sex, diabetes mellitus, hypertension, atrial fibrillation, dyslipidemia, smoking, education, insurance, time from stroke onset to admission interval, mRS before the stroke, baseline NIHSS score, NIHSS score 24h after EVT, the side of the brain where the stroke occurred, stroke cause, and TICI score of 2b or 3*.

### Subgroup Analyses

**Figure 3:**
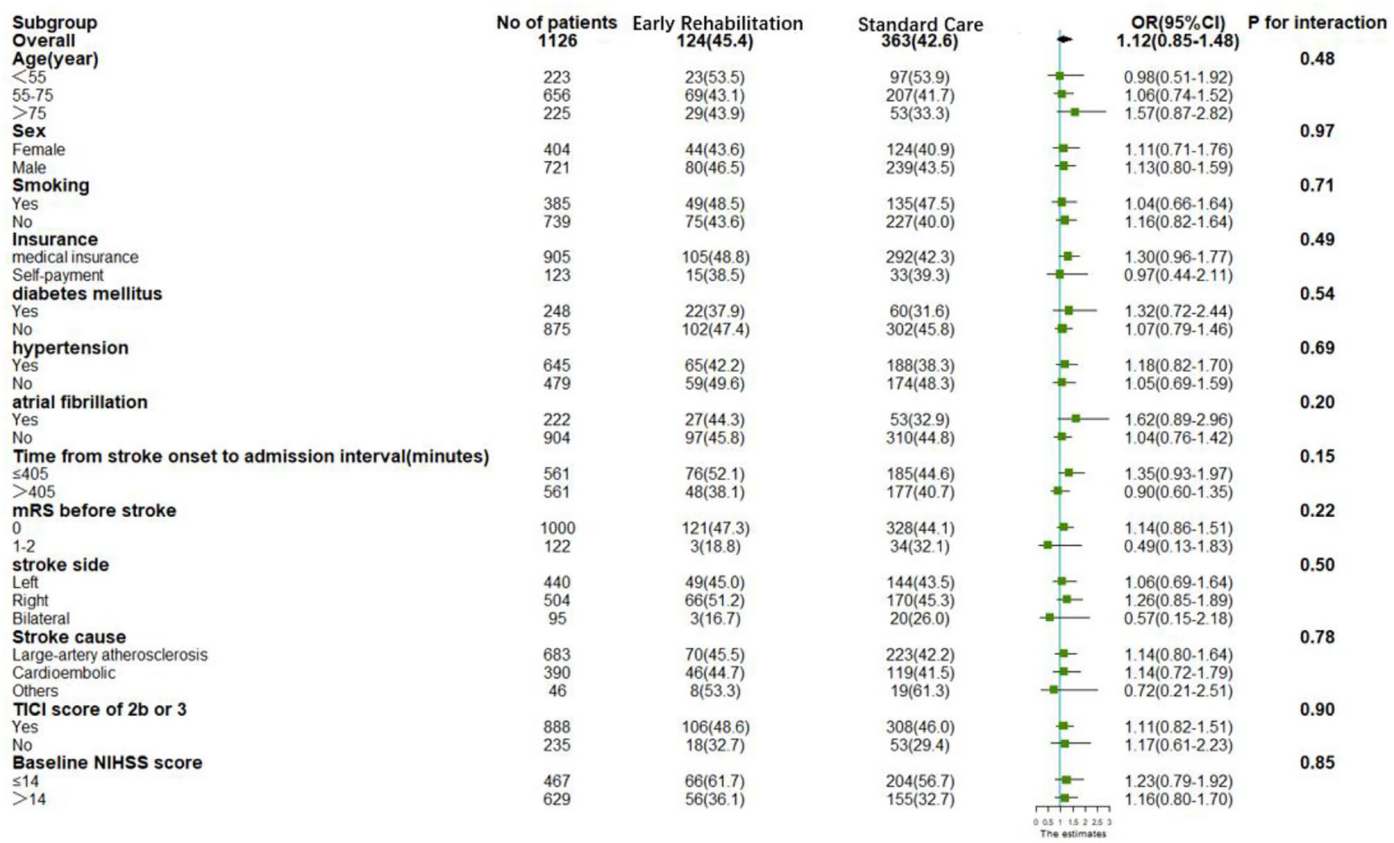
Subgroup analysis of the primary outcome. The forest plot shows the differences in odds ratio for primary outcome (modified Rankin Scale [mRS], 0–2) at 90 days in subgroups. Adjusted for age, sex, diabetes mellitus, hypertension, atrial fibrillation, dyslipidemia, smoking, education, insurance, time from stroke onset to admission interval, mRS before the stroke, baseline NIHSS score, NIHSS score 24h after EVT, the side of the brain where the stroke occurred, stroke cause, TICI score of 2b or 3. OR, odds ratio; CI, confidence interval; mRS, modified Rankin Scale; TICI, thrombolysis in cerebral infarction; NIHSS, National Institute of Health Stroke Scale.

The results of subgroup analyses on the primary outcome are shown in the forest plot in Figure 3. There was no heterogeneity in the effects of early rehabilitation on the favorable functional recovery (mRS 0–2) at 90 days between subgroups classified by age, sex, smoking, insurance, medical history, time from stroke onset to admission interval, mRS before the stroke, the side of the brain where the stroke occurred, stroke cause, TICI score of 2b or 3, and baseline NIHSS score. There were no significant interactions.

## Discussion

Using a multicenter inpatient database in China, this study is the first to analyze the long-term (90 days) effects of early rehabilitation in patients with AIS after EVT in real-world clinical practice. The main finding of this study demonstrated that patients with stroke receiving early rehabilitation had a lower death rate than those who did not receive rehabilitation during hospitalization. However, these clinically meaningful effects of early rehabilitation did not show on functional outcomes at 90 days. Although the proportion of favorable functional outcome (mRS 0–2) at 90 days was higher in the early rehabilitation group, no significant difference was recorded between the two groups in patients with LVO treated with EVT. In a randomized controlled study in A Very Early Rehabilitation Trial for stroke (AVERT), Bernhardt used subgroup analyses in patients receiving recombinant tissue plasminogen activator and found similar results, namely, no significantly favorable functional outcome at 3 months was observed in early rehabilitation group (10).

Another finding of this study is that patients with stroke receiving early rehabilitation had a longer length of hospital stay than those who receive standard care during hospitalization. Length of hospital stay is the largest determinant of the direct cost for stroke care and is predominately determined by medical complications (23). This study revealed an interesting phenomenon that the early rehabilitation group had a longer length of hospital stay but without increased costs. From the viewpoint of decision-makers who are faced with scarce resources, the economic justification for implementing rehabilitation is equally important as clinical efficacy for rehabilitation. In this study, the medical cost during hospitalization was lower in the early rehabilitation group, although no significant difference was recorded between groups, which was consistent with the previous studies. Teo suggested that very early rehabilitation is potentially cost-saving and likely to be cost-effective in comparison with standard care alone (24).

The main limitation of this study is the lack of unified records of rehabilitation protocol in the database. The rehabilitation data were based on self-reports from each center. The array of rehabilitation services delivered to patients with stroke in China is highly personalized and heterogeneous, varying in the duration, intensity, and type of interventions. The selection bias due to varied rehabilitation protocols provided across participating centers could not be neglected. Delivering rehabilitation in a standardized manner in all centers may address this issue. Some trials reported detailed protocols of the rehabilitation program, in which a unified training program was provided for the therapist to ensure consistency in rehabilitation treatment (25–27). Use of the template for the intervention description and replication (TIDieR) checklist (28) or the Rehabilitation Treatment Specification System (29) will improve the reliability and the reproducibility of trials. However, it would be complex and time-consuming. Rehabilitation is not as easily controlled as pharmacological interventions. The rehabilitation protocol changes dynamically depending on the patient's functional status. More improvements are needed in the future studies.

The second limitation is that to minimize the effects on results, we exclude the patients without rehabilitation data and lost to follow-up, which may generate a selection bias since patients without rehabilitation data perhaps because they did not receive any rehabilitation, and those lost to follow-up possibly non-compliant with rehabilitation. However, we find that the demographic characteristics of two groups were not significantly different. Although there are differences in age, education level, prestroke mRS, and residual stenosis between two groups. In multivariate logistic regression analyses for outcomes, after adjusting for potential confounders, the results showed that there was no effect of potential confounders on outcomes (Table 3), which means that the two groups were comparable.

The third limitation is that patients who initiated early rehabilitation with less abnormal mRS prestroke, lower residual stenosis, and other variables that might not have been registered. We used multivariate logistic regression analyses to examine whether these variables have effect on outcomes. In multivariate logistic regression analyses for outcomes, after adjusting for potential confounders [including mRS before stroke, TICI score of 2b or 3, and other variables, which might influence outcomes according to previous studies (19, 21, 22)], the results showed that there was no effect of potential confounders on outcomes (Table 3). This limitation may be resolved after expanding sample in further data collection.

Another limitation is that the study population could not reflect the general population with stroke as only patients with LVO after EVT were included, which means that the sample was relatively older (64.7 ± 12.2), and the proportion of patients with moderate to severe stroke (50% NIHSS≧16) was greater than a standard stroke population. This difference limits the generalizability of the results in the general stroke population. Other studies also observed negative results among patients with moderate stroke (NIHSS score ranging from 7 to 9) (10, 30). Hence, the neutral results at 90 days in this study would be apprehensible.

## Conclusion

To conclude, this study showed that patients with stroke receiving rehabilitation had a lower death rate than those who did not receive rehabilitation during hospitalization. However, these clinically meaningful effects of rehabilitation did not show on functional outcome at 90 days in patients with LVO after EVT.

## Data Availability Statement

The raw data supporting the conclusions of this article will be made available by the authors, without undue reservation.

## Ethics Statement

The studies involving human participants were reviewed and approved by the IRB of Beijing Tiantan Hospital Affiliated to Capital Medical University. The patients/participants provided their written informed consent to participate in this study.

## Author Contributions

YH and XN contributed to research project conception and design, statistical analysis, and manuscript writing. TH, XQ, ZC, WD, YW, and XL: data collection. YL: research project conception and design, statistical analysis review and critique, and manuscript review and critique. All authors contributed to manuscript revision, read, and approved the submitted version.

## Funding

This study was supported by the National Key R&D Program of China (2016YFC1307301, 2018YFC1312402) and National Natural Science Foundation of China (81820108012).

## Conflict of Interest

The authors declare that the research was conducted in the absence of any commercial or financial relationships that could be construed as a potential conflict of interest.

## Publisher's Note

All claims expressed in this article are solely those of the authors and do not necessarily represent those of their affiliated organizations, or those of the publisher, the editors and the reviewers. Any product that may be evaluated in this article, or claim that may be made by its manufacturer, is not guaranteed or endorsed by the publisher.
